# An inexpensive and easy-to-make customized antibiotics mix for mycobacterium culture

**DOI:** 10.12688/f1000research.14467.1

**Published:** 2018-04-27

**Authors:** Ashwani Kesarwani, Puja Nagpal, Alaknanda Mishra, Rana Zaidi, Pramod Upadhyay

**Affiliations:** 1 National Institute of Immunology, New Delhi, 110067, India; 2 Department of Biochemistry, Jamia Hamdard, New Delhi, 110062, India

**Keywords:** mycobacterium culture, antibiotics, customized antibiotics mix

## Abstract

The cultivation of mycobacteria often requires the use of several antibiotics to limit the growth of other rapidly growing micro-flora present in the growth medium. This antibiotic cocktail is one of the most expensive reagents required for mycobacterium culture. Here we present a customized antibiotics mix that is easy to prepare at a fraction of the cost of the commercially available antibiotic mixture that protects against transient flora, which are normally present in lungs, without affecting mycobacterial colony number.

## Introduction

Mycobacteria are slow-growing organisms ( Lambrecht *et al.*, 1988); to obtain visible colonies their culture has to continue for several days. Often the fluid to be examined for mycobacterium contains many other microflora and growth of this microflora has to be limited to allow the mycobacterium to grow. For this purpose, a cocktail of antibiotics is used in the culturing of mycobacterium. This antibiotics cocktail is one of the most expensive reagents (INR 4500 per pack, sufficient for 3 l of media) required for mycobacteria culture. We have formulated a Customized Antibiotics Mix (CAM), which is a mixture of antibiotics, to inhibit or reduce the growth of other micro-organisms.

The CAM has the following antibiotic components. Polymyxin B is a mix of polymyxin B1 and B2, basic polypeptides obtained from strains of *Bacillus polymyxa*. Polymyxin B acts as a bactericidal against all Gram-negative bacilli except *Proteus* and *Neisseria* genera by binding with the cell membrane and increasing its permeability, changing its structure and causing a higher uptake of water, ultimately leading to cell death ( Cardoso *et al.*, 2007).

Amphotericin B is an antifungal drug first prepared from *Streptomyces nodosus* in 1955 ( Donovick *et al.*, 1955). This drug is also used to treat aspergillosis, blastomycosis, coccidioidomycosis, cryptococcosis and candidiasis. Amphotericin B causes the fungal cell to leak monovalent ions by binding with ergosterol, an integral part of fungal cell membrane, eventually causing fungal cell death ( Mesa-Arango *et al.*, 2012; O’Keeffe *et al.*, 2003).

Nalidixic acid is a very weak organic acid used for the treatment of bacterial urinary tract infection such as *Escherichia coli*, *Enterobacter*, *Klebsiella*, *Proteus* and *Shigella*. It is a synthetic quinolone antibiotic, which is a group of antibiotics that inhibit bacterial growth by selectively blocking the DNA replication of these bacteria. Hence, it is also used for the study of regulation of bacterial division ( Pommier *et al.*, 2010).

Trimethoprim is a synthetic antibacterial drug mainly used for the treatment of bladder infections. It typically targets species such as *Escherichia coli*, *Proteus mirabilis*, *Klebsiella pneumoniae* and *Enterobacter* species. This drug inhibits the DNA synthesis of bacteria, hindering the reduction of dihydrofolic acid to tetrahydrofolic acid, which is a key precursor in the thymidine kinase pathway ( Brogden *et al.*, 1982).

Azlocilin is a semisynthetic broad-spectrum antibiotic used against a number of Gram-positive and -negative bacteria. Azlocilin weakens the cell wall of the bacteria by binding to the penicillin-binding protein located inside the bacterial cell wall, which in turn inhibit the crosslinking of peptidoglycan ( Sanders, 1983).

All of these aforementioned reagents are among those commonly used in antibiotics formulations for treating infections.

## Methods

### Preparation of CAM

For the preparation of CAM, the aforementioned commercially available antibiotic formulations were procured and their working stocks were prepared in water. From the working stock, the calculated amount volume of antibiotics were mixed. The details for the preparation of working stock and the final volume used for the preparation of 5 l Middlebrook 7H11 agar medium, sufficient for around 200 culture plates, are given in Table 1.

**Table 1.** Antibiotics and their concentration used for the preparation of CAM.

Serial number	Antibiotics/Name of commercial formulation	Concentration of antibiotics per pack	Concentration of working stock solution	Required amount antibiotic for 5 l agar media	Cost (INR) per pack * (for 5 l agar media)	Volume of working stock used for 5 l media
**1.**	Polymyxin B/POLY-B™	~500,000 IU per 580 mg	3.5 mg/1000 µl	30,000 IU	1600/pack (10)	1000 µl
**2.**	Amphotericin B/AMPHOTRET™	50 mg per pack	10 mg/1000 µl	3,000 µg	300/pack (60)	300 µl
**3.**	Nalidixic acid/GramoNeg®	500 mg per tab	1 tab/10 ml	12,000 µg	25/10Tab (2.5)	240 µl
**4.**	Trimethoprim/Bactrim®	160 mg per tab	1 tab/10 ml	3,000 µg	20/10 Tab (2)	187.5 µl
**5.**	Azlocilin/Azenam	1 g per pack	10 mg/1000 µl	3,000 µg	650/pack (6.5)	300 µl

*65 INR ≈ 1 USD.

Typically, one tablet each of nalidixic acid (GramoNeg®; Best laboratories Pvt. Ltd.) and Trimethoprim (Bactrim®; Piramal Enterprises Limited) tablets were dispersed in 10 ml water and kept on rocker shaker for 15–20 mins, the mixture was then centrifuged at 600 *g* for 10 min. Supernatant was aspirated and used for the preparation of the CAM.

Required amounts (shown in Table 1) of Polymyxin B (POLY-B ^TM^; Samarth Life Sciences Pvt. Ltd.), Amphotericin B (AMPHOTRET ^TM^; Bharat Serum and Vaccine Limited) and Azlocilin (Azenam; ARISTO Pharmaceuticals Pvt. Ltd.) were weighed and dissolved in water. The CAM was prepared by mixing appropriate volumes of working stock solutions and was filtered through a 0.2-µm syringe filter.

### Preparation of agar plates

A total of 105 g Difco ^TM^ Mycobacteria 7H11 Agar (BD Biosciences, USA) was suspended in 4,500 ml water containing 25 ml glycerol. The medium was swirled on a hot magnetic plate to obtain a smooth suspension and autoclaved at 121°C for 15 min.

The medium was allowed to cool to 50–55°C in aseptic conditions. In the meantime, 25 g bovine serum albumin, 10 g dextrose, 15 mg catalase and 4.25 g sodium chloride (all Himedia, India) were dissolved in 500 ml water and filtered through a 0.2-µm filter. Next, 250 µl oleic acid (Himedia, India) was then added aseptically. This mix is commonly known as OADC ( oleic acid, albumin, dextrose and catalase).

Ten vials of commercial BBL ^TM^ MGIT ^TM^ PANTA ^TM^ (BD-Panta) antibiotic mixture (Becton, Dickson and company, USA) was then added to 5 l media. In another preparation of media, the BBL ^TM^ MGIT ^TM^ PANTA ^TM^ was replaced with the CAM, formulated as aforementioned.

### Mice immunization with BCG

To compare the efficacy of the two antibiotic mixes, eight female B57BL/6J mice of 4–6 weeks age, weighing 20–25 g were immunized with *Mycobacterium bovis* (BCG) by the aerogenic route to establish around 1,000–2,000 bacilli of BCG in each mouse ( Bhaskar & Upadhyay, 2003). Mice were house in ventilated cages and fed with autoclaved acidified water and irradiated food *ad libitum* and were kept in 12 h light and 12 h dark conditions.

Use of animals in this investigation was approved by the Institutional animal ethical committee of National Institute of Immunology, New Delhi (IAEC#354/14).

### Estimation of bacilli load

At every time point of day 1, 7, 14 and 30 post-immunization, two immunized mice were euthanized by an overdose of Ketamine and Xylazine given intraperitoneally. Typically, 35 mg ketamine and 3.5mg xylazine in 350 µl saline per mouse was used to euthanize a mouse.

Their lung and spleen were isolated aseptically and homogenized in 1 ml PBS using a tissue homogenizer (Polytron PT 1600E, Germany) at 30,000 rpm for 40 s. The homogenized mix was diluted 5 times in PBS and 100 µl diluted mix was spread on the aforementioned agar plates in triplicate.

At every time point the tissue homogenates were plated on media plates prepared using CAM and commercial BD-Panta antibiotic mixture.

Plates were incubated at 37°C for 4 weeks.

### Statistical analysis

GraphPad Prism 7 software was used to calculate p-values by two-way ANOVA.

## Results

**Figure 1.**
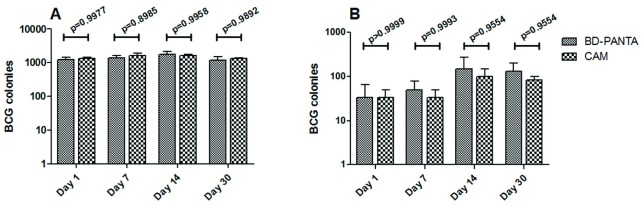
*Mycobacterium bovis* (BCG) colonies in the lung ( **A**) and spleen ( **B**) after immunization. At each indicated time point, the lung and spleen were isolated from immunized mice followed by single-cell suspension preparation and plating on 7H11 agar plates with Customized Antibiotic Mix (CAM) or BD-PANTA. The number of colonies were counted and compared between the two groups.

After incubation, BCG colonies present on plates were counted, the results of which are shown in Figure 1. The data ( Dataset 1) confirm that on prepared agar plates BCG was able to selectively grow from a complex micro-flora of the lung and spleen. Similar number of BCG colonies were observed on CAM plates and BD-PANTA plates; the differences between the two were statically insignificant ( Figure 1).

Number of BCG bacilli colony forming units (CFUs) on each plate from each experimental group The data show CFUs on each plate along with calculated bacilli load of the tissue. Click here for additional data file. Copyright: © 2018 Kesarwani A et al. 2018 Data associated with the article are available under the terms of the Creative Commons Zero "No rights reserved" data waiver (CC0 1.0 Public domain dedication).

## Conclusions

The cost of above discussed customized antibiotics mix for preparing 5 l of agar media was around 80 INR (around 1.25 USD) which is almost 1/100 ^th^ of the cost of commercially available antibiotic formulation for the purpose. This antibiotic mix is highly economical, easy to prepare and can significantly reduce the total cost involved in mycobacterium culture.

## Data availability

The data referenced by this article are under copyright with the following copyright statement: Copyright: © 2018 Kesarwani A et al.

Data associated with the article are available under the terms of the Creative Commons Zero "No rights reserved" data waiver (CC0 1.0 Public domain dedication). 

**Dataset 1. Number of BCG bacilli colony forming units (CFUs) on each plate from each experimental group.** The data show CFUs on each plate along with calculated bacilli load of the tissue. http://dx.doi.org/10.5256/f1000research.14467.d201182 ( Kesarwani *et al.*, 2018).
