# Supplementary material for: An inexpensive and easy-to-make customized antibiotics mix for mycobacterium culture
Source: F1000Res. 2018 Apr 27;7:507. [Version 1] doi: 10.12688/f1000research.14467.1 (PMC5989142; doi:10.12688/f1000research.14467.1)
Supplement: Peer review history

# Peer review: Referee response for version 1

**DOI:** 10.5256/f1000research.15748.r34517

**Published:** 2018-06-04

**Authors:** Bhupendra N. Singh

**Affiliations:** 1 Division of Microbiology, CSIR-Central Drug Research Institute, Lucknow, India

The authors have addressed a very pertinent issue of growing mycobacterial culture using a customized cocktail of commonly available antibiotics instead of commercially available expensive antibiotics (PANTA), presently in use. They have performed whole experiments in a very simple way and succinctly described the methods with appropriate conclusions. Since the findings obtained using CAM were comparable to the commercially available PANTA, this can be really useful to researchers in TB field.

 Bypassing the routine culture and BACTEC methods authors tested CAM in animal based study, which gave ample scope to new mixture of antibiotics to eradicate any prevailing contamination carried forwarded through organ homogenate. However, a control carrying only organ homogenates on the culture media plates without any antibiotic mix would have given fair idea of prevailing microbial contamination and allowed authors to assess how effectively CAM exterminated the microbial flora under the experimental conditions used by authors.

I have read this submission. I believe that I have an appropriate level of expertise to confirm that it is of an acceptable scientific standard.

# Peer review: Referee response for version 1

**DOI:** 10.5256/f1000research.15748.r33548

**Published:** 2018-05-09

**Authors:** Amit Singh

**Affiliations:** 1 Department of Microbiology and Cell Biology, Centre for Infectious Disease Research, Indian Institute of Science, Bengaluru, Karnataka, India

This is a very simple paper based on common microbiological techniques. Authors have taken a few common antibiotics and made a cocktail which is cost-effective and gave results comparable to the existing commercially available antibiotics (PANTA). Experiments are planned well, appropriately described, and conclusions are justified. The comparable findings obtained using this highly cost-effective formulation relative to commercially available formulations can be of great help to researchers in TB field.

 To check the reliability of the new formulation, authors have conducted animal experiments and enumerated bacillary load in the infected organs such as lungs and spleen. Authors detected no significant difference between commercial and the new mixture of antibiotics on the growth potential of the BCG strains isolated from the infected lungs. The statistical analysis was done using two-tailed ANOVA, which is most appropriate. 

 While experiments are reliable and results are presented well, I would have liked a control wherein authors plate organ homogenates on the culture media plates without any antibiotic mix. This would have confirmed that the normal microflora present in these organs are interfering with the outgrowth of BCG cells under the experimental conditions used by the authors. This way authors can bolster the efficacy of the antibiotic combination in reducing contamination in the mycobacterial cultures.

I have read this submission. I believe that I have an appropriate level of expertise to confirm that it is of an acceptable scientific standard.
